# The Role of Hearing Aids in Improving Dual‐Task Gait Performance in Older Adults With Presbycusis: A Cognitive and Motor Analysis

**DOI:** 10.1002/brb3.70114

**Published:** 2024-10-31

**Authors:** Emre Soylemez, Tugce Gurel Soylemez, Aydin Sinan Apaydin, Zuhal Koc Apaydin, Murat Yasar

**Affiliations:** ^1^ Department of Otorhinolaryngology Karabuk University Karabuk Turkiye; ^2^ Clinical Audiologist Karabuk Training and Research Hospital Karabuk Turkiye; ^3^ Department of Neurosurgery Karabuk University Karabuk Turkiye; ^4^ Department of Psychiatry Karabuk University Karabuk Turkiye; ^5^ Department of Otorhinolaryngology Kastamonu University Kastamonu Turkiye

**Keywords:** dual task, hearing aids, hearing loss, presbycusis, working memory

## Abstract

**Background:**

Cognitive decline is a common challenge faced by older individuals with presbycusis; their performance on dual‐task (DT) activities is generally lower compared to those without hearing loss. However, the influence of hearing aids on nonauditory cognitive decline in this population remains unclear. This study aims to investigate the effect of hearing aids on nonauditory DT performance in older adults with presbycusis.

**Material and Methods:**

This study included older people with presbycusis who used hearing aids (P&HA group), those with presbycusis who did not use hearing aids (PoHA group), and a control group of healthy adults. Forward and backward digit span tests and timed up and go test (TUG) were administered to all individuals. TUG and motor and cognitive (forward and backward digit span) tasks were applied simultaneously to evaluate the participants' DT performance.

**Results:**

The study comprised 60 individuals with presbycusis (19 females, 41 males; mean age: 73.23 ± 6.49 years) and 30 healthy adults (15 females, 15 males; mean age: 35.93 ± 8.57 years). Healthy adults performed better than the P&HA and PoHA groups in all the administered tests (*p* < 0.05). There was a moderate negative relationship between the severity of hearing loss and the forward and backward digit span test performances (*p* < 0.05). The P&HA group performed better than the PoHA group on the DT cognitive forward and backward digit span tests.

**Conclusion:**

The use of hearing aids and their proper fitting are important not only for improving communication skills and reducing listening effort but also for supporting nonauditory cognitive functions, minimizing the risk of falls during DT activities, and enhancing the overall quality of life.

## Graphical abstract

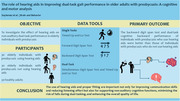

## 1 Introduction

Presbycusis is the third‐most common chronic disorder seen in older individuals, after arthritis and hypertension (Bess and Hedley‐Williams 2001; Miranda et al. 2007). Almost half of individuals aged 65 years and over have presbycusis (Goman and Lin 2016). The prevalence of presbycusis is expected to rise as advancements in medicine and healthcare systems extend life expectancy. Therefore, presbycusis, which lacks medical or surgical treatments, will increase the burden on public healthcare systems in the future.

Internal and external factors play crucial roles in the formation and development of presbycusis (Aldè et al. 2023; Yang, Schrepfer, and Schacht 2015). Exposure to noise, the use of ototoxic medications, exposure to chemicals, and lack of nutrition can independently cause hearing loss or modulate the external factors associated with presbycusis (Yang, Schrepfer, and Schacht 2015). Internal factors include genetic predisposition, hypertension, diabetes mellitus, and other metabolic and systemic diseases. Genetic factors determine the characteristics that affect the structure, function, and resilience of the cells in the inner ear, shaping individuals' susceptibility to hearing loss during the aging process (Aldè et al. 2023).

Introversion, family problems, low self‐esteem, isolation, depression, and irritability due to communication disorders are frequently observed in older persons with presbycusis. In addition, sensory disorders, such as balance and gait issues, and cognitive disorders, such as cognitive decline and memory problems, are frequently observed in older adults with presbycusis (Li et al. 2013; Mudar and Husain 2016). Older adults must use more cognitive capacity to compensate for sensory impairments (Li and Lindenberger 2002). Moreover, hearing/auditory processing and balance maintenance tasks co‐occur in daily life. Since the cognitive capacities of these individuals also decrease, competition arises regarding the use of cognitive capacity during dual‐task (DT). This performance loss occurring in one or both tasks is called dual‐task cost (DTC) (Li and Lindenberger 2002; Wollesen et al. 2018). DT is often assessed using motor and cognitive tasks administered simultaneously with a walking or balance task. In DT situations, individuals prioritize walking or balance tasks for physical safety. Due to this situation, known as the “posture‐first” strategy, DTC is determined by the deterioration in walking performance (Shumway‐Cook et al. 1997).

The cause of cognitive decline in older people with presbycusis is unclear. However, two hypotheses are considered (Mudar and Husain 2016; Wunderlich et al. 2021). First, cognitive decline and hearing loss can occur due to widespread deterioration due to aging. The other is that, due to hearing loss, more capacity is allocated to auditory processing, and momentary cognitive impairments occur (Mudar and Husain 2016; Wunderlich et al. 2021). Older individuals with presbycusis require more cognitive resources to understand speech, analyze context, and recall previous phonological information. Thus, less resources are allocated to other nonauditory processes (Monzani et al. 2022).

Hearing aids amplifying sounds improve auditory function, reduce listening fatigue, and increase awareness (Monzani et al. 2022). Studies in the literature have primarily focused on the effect of hearing aids on listening effort/fatigue or the effect of hearing loss on DT performance (Monzani et al. 2022; Wollesen et al. 2018; Wunderlich et al. 2021). To the best of our knowledge, no study has evaluated the effect of DT on nonauditory motor and cognitive tasks in elderly individuals using hearing aids. Knowing the effect of hearing aids on nonauditory DT in elderly individuals with presbycusis is important to understand the performance of these individuals in motor and cognitive tasks in their daily lives and to evaluate the potential benefits of hearing aids in these tasks. Therefore, this study aims to investigate the effect of hearing aids on nonauditory DT performance in elderly individuals with presbycusis.

## 2 Materials and Methods

### 2.1 Ethical Situation

Permission was received for this study from the university's clinical research ethics committee (Decision no: 2024‐KAEK‐28). The study was started after ethical approval. Verbal and written permission was obtained from all participants included in the study.

### 2.2 Participants and Procedure

Elderly individuals (age > 65 years) who applied to the otorhinolaryngology outpatient clinic with complaints of hearing loss were included in this study. Anamnesis was taken from the participants, and an otoscopic examination was performed. The patients were referred to the audiology clinic, and pure tone audiometry and immitansmetric tests were performed. According to the evaluations, 60 elderly individuals diagnosed with presbycusis were included in the study (Kim and Chung 2013). The education level of these individuals, hearing aid usage status (yes/no), duration of hearing aid use (years), and number of hearing aids (unilateral/bilateral) were noted. Elderly individuals with presbycusis were divided into two groups according to their hearing aid usage status: hearing aid users (P&HA group) and non‐users (PoHA group). Thirty healthy individuals, aged between 21 and 50, were included in the study as a control group. Participants were excluded if they had a traumatic head injury/surgery, vertigo (such as BPPV, Meniere's disease, vestibular neuritis or labyrinthitis), eardrum perforation, conductive or mixed hearing loss, neurological and uncontrollable systemic disease (hypertension, diabetes mellitus, and cardiovascular disease), visual impairment, cognitive impairment [Standardized Mini‐Mental Exam: SMME < 24)], and musculoskeletal injuries/diseases.

### 2.3 Audiological Evaluation

All individuals underwent pure tone audiometry testing in a soundproof booth using a clinical audiometer (AC40; Interacoustics, Denmark). The air conduction thresholds of the participants were tested at octave frequencies between 250 and 8000 Hz using TDH‐39 supra‐aural headphones, while the bone conduction thresholds were assessed at octave frequencies between 250 and 4000 Hz using a Radio‐ear B‐71 bone vibrator. The pure tone air conduction average (PTA) was calculated based on the average of the air conduction thresholds at 500, 1000, 2000, and 4000 Hz. The Global Burden of Disease approach was used to classify hearing loss, with a PTA greater than 20 dB considered indicative of hearing loss (Stevens et al. 2013).

### 2.4 DT Performance

DT performance was determined by the time up and go (TUG) test and simultaneously applied motor and cognitive tasks. Participants sitting on the chair were asked to walk quickly on a 3‐m track and return from the endpoint to sit on the chair again. The participant's time to complete the course was measured with a stopwatch. In this way, single‐task TUG performance was determined.

To evaluate DT motor performance (DT_motor_), participants were asked to carry a tray with a glass filled with water during the TUG. The participant's DT_motor_ time was measured with a stopwatch.

A computerized visual digit span test was used to give participants a cognitive task during DT. A computerized visual digit span test was presented to the participants using PowerPoint software, with one digit appearing every second. The test started with a sequence of three numbers. Participants were asked to keep the numbers in mind and repeat them at the end of the test. With each correct answer, the number sequence was increased by one. The test was terminated in case of two consecutive incorrect repetitions, and the participants' maximum forward digit number was determined. The same method was used to determine the participants' maximum number of backward digit spans. However, for the backward digit span, participants were asked to keep in mind and repeat the numbers appearing on the screen in reverse order.

TUG and forward (DT Cognitive_forward_) and backward (DT Cognitive_backward_) digit span tests were applied together to evaluate DT cognitive performance. Participants were presented with a sequence of numbers as long as they could remember. However, the participants were asked to apply the TUG after the number sequences were finished and to repeat the number sequence when they sat on the chair. Participants' DT Cognitive_backward_ and DT Cognitive_forward_ times were measured with a stopwatch.

The following formula was used to evaluate DTC in participants (Kelly and Janke 2010).

$$\frac{\pm \left(\text{Single Task}-\text{Dual Task}\right)}{\text{Dual Task}}\times 100=\text{Dual Task Cost}\;\left(\mathrm{DTC}\right)\%$$

### 2.5 Statistical Analysis

IBM SPSS 21 software was used for statistical analysis. Normality distribution was checked with the Shapiro–Wilk test. Normally distributed data were presented as mean ± SD, and non‐normally distributed data were presented as median (min–max). One‐way ANOVA or Student's *t*‐test was used to compare parametric data. Kruskal–Wallis test or Mann–Whitney *U* test was used to compare nonparametric data. Categorical variables were evaluated with the chi‐square test. Since the data were not normally distributed, the Spearman correlation test was used to evaluate the relationship between groups. The range of ±0.3 to ±0.7 was accepted for a moderately strong correlation coefficient. In all statistical analyses, *p* < 0.05 was accepted as the level of statistical significance.

## 3 Results

The mean age of the P&HA group was 73.17 ± 5.64 (65–82), and the mean age of the PoHA group was 73.30 ± 7.34 (65–94). There was no difference in age between the two groups (*p* = 0.959). The mean age of the control group was 35.93 ± 8.57 (21–50). Of the elderly individuals in the P&HA group, 10 (33.3%) were female and 20 (66.7%) were male. Of the elderly individuals in the PoHA group, 9 (30.0%) were female and 21 (70.0%) were male. Of the individuals in the control group, 15 (50.0%) were female and 15 (50.0%) were male. There was no difference between the groups in terms of gender (*p* = 0.231).

Of the individuals in the P&HA group, 4 (13.3%) were primary school graduates, 8 (26.7%) were secondary school graduates, 16 (53.3%) were high school graduates, and 2 (6.7%) were university graduates. Of the individuals in the PoHA group, 9 (30.0%) were primary school graduates, 9 (30.0%) were secondary school graduates, 10 (33.10%) were high school graduates, and 2 (6.7%) were university graduates. There was no difference between the groups in terms of educational status (*p* = 0.338).

**FIGURE 1:**
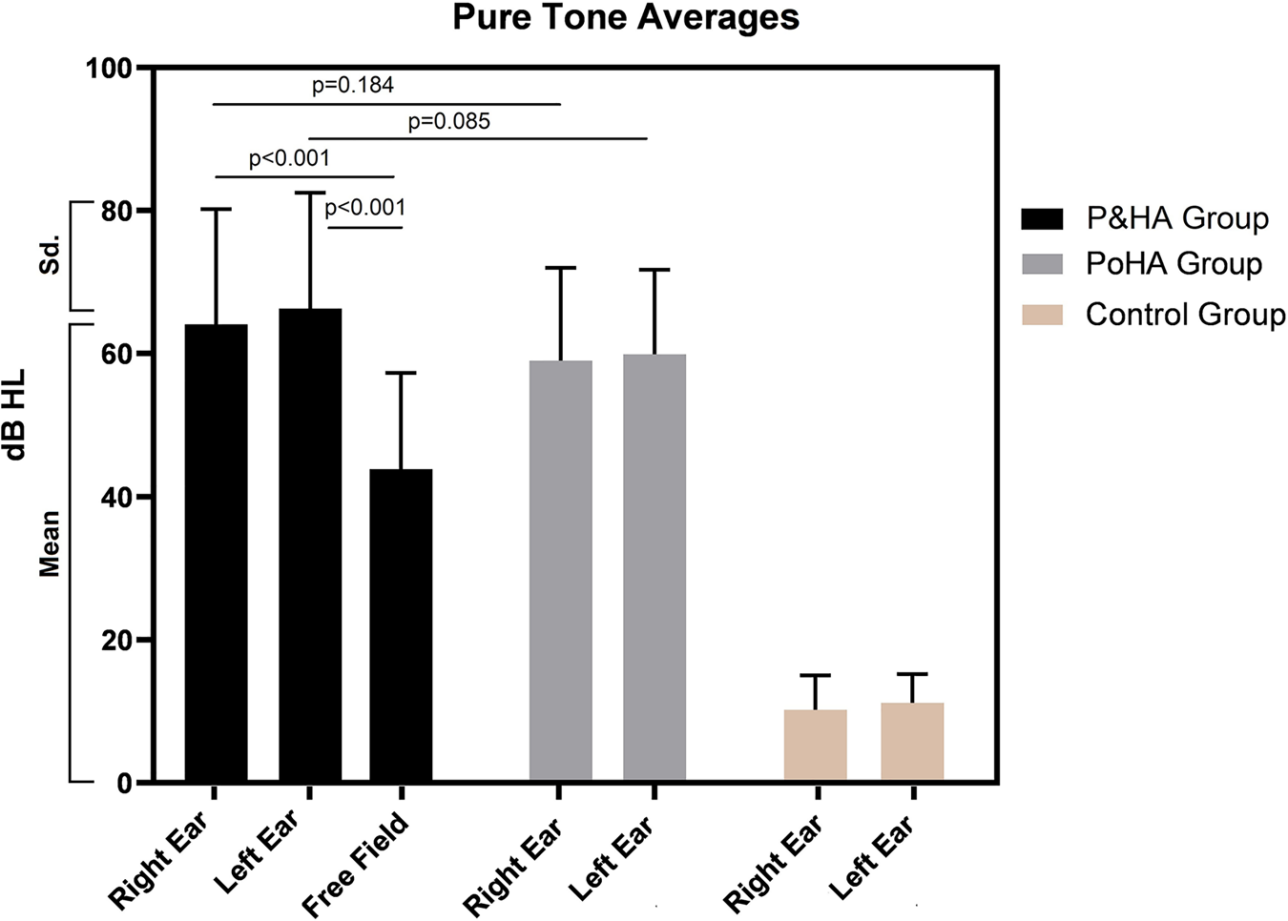
PTA values by groups. Free field audiometry in the P&HA group was performed with a hearing aid. Good ear PTA values of individuals using bilateral hearing aids are presented.

Twenty‐six (86.7%) of the individuals in the P&HA group were using unilateral hearing aids, four (13.3%) were using bilateral hearing aids, and the median duration of hearing aid use was 5.0 (1.0–21.0) years. There was no difference in PTA between the P&HA group and the PoHA group (*p* > 0.05). PTA with hearing aid performed in the free field of the P&HA group was better than PTA without hearing aid (*p* < 0.05). PTA values according to groups are presented in Figure 1.

**FIGURE 2:**
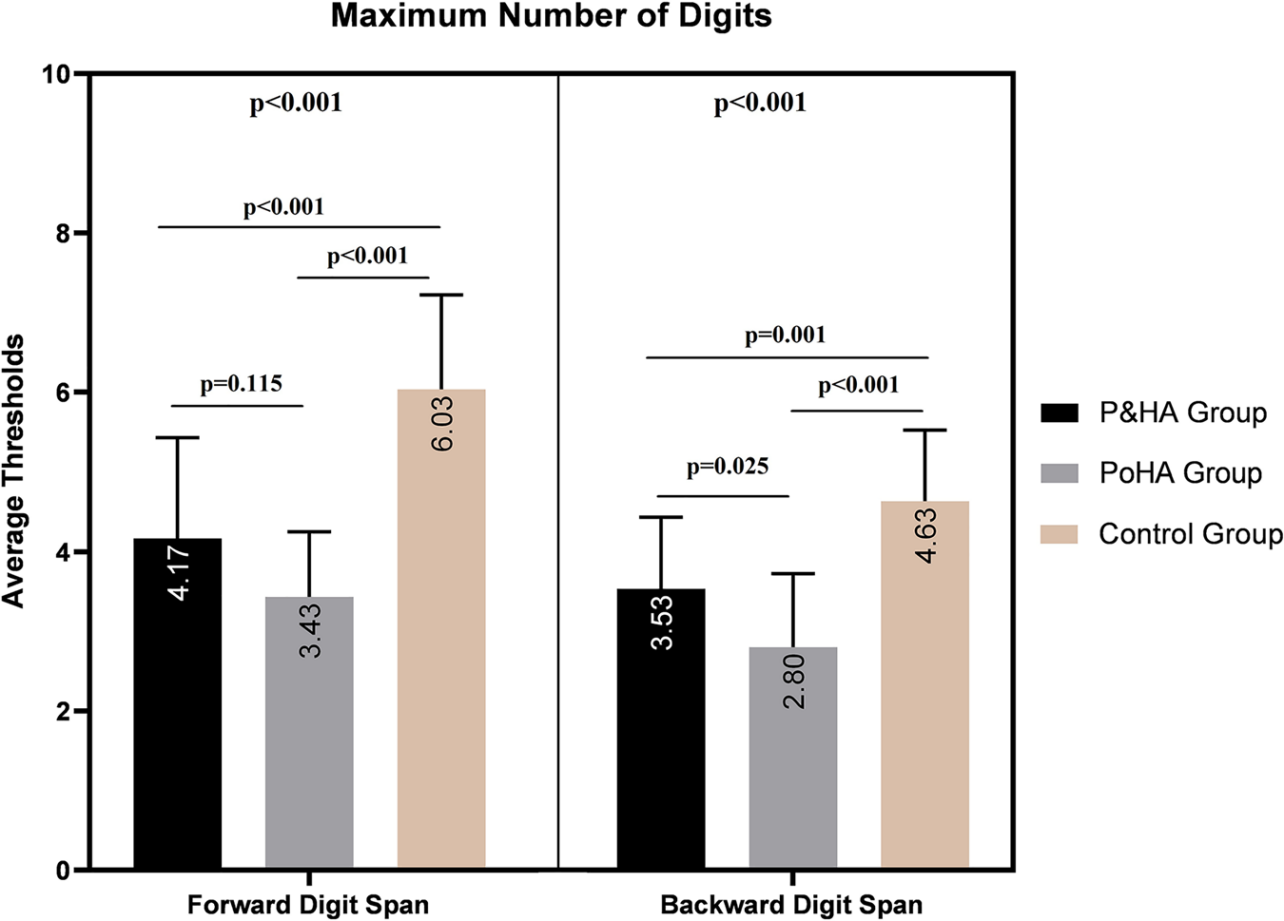
Forward and backward maximum digit span numbers according to groups.

The maximum forward and backward digit span tests of the control group were better than the P&HA and PoHA groups (*p* < 0.05). There was no difference between the P&HA and PoHA groups in terms of the maximum forward digit span test (*p* > 0.05). However, the maximum backward digit span test of the P&HA group was better than the PoHA group (*p* < 0.05). Forward and backward maximum digit span tests according to groups are presented in Figure 2.

The P&HA group had no relationship between the duration of hearing aid use and maximum forward and backward digit span tests (*p* = 0.364 and 0.133, respectively). However, there was a moderate negative relationship between the participants' right and left ears PTA values and the maximum forward and backward digit span tests (*p* < 0.05). The relationship between right and left ears PTA and forward and backward maximum digit span numbers is presented in Table 1.

**TABLE 1:** Relationship between right and left ears PTA and forward and backward maximum digit span numbers.

		Correlation coefficient (*p* value)	
		Maximum forward digit span	Maximum backward digit span
	Median (min–max)	4 (2–8)	4 (2–6)
Right ear, *n* = 90	51.25 (3.75–97.50)	−0.49 (*p* < 0.001)	−0.48 (*p* < 0.001)
Left ear, *n* = 90	52.50 (3.75–101.25)	−0.54 (*p* < 0.001)	−0.47 (*p* < 0.001)

The single task TUG, DT_motor_, DT Cognitive_forward_, and DT Cognitive_backward_ performance of the control group was better than the P&HA and PoHA groups (*p* < 0.001). DT Cognitive_backward_ performance of the P&HA group was better than that of the PoHA group (*p* < 0.001). However, there was no difference between the P&HA and PoHA groups in terms of single task TUG, DT_motor_, and DT Cognitive_forward_ (*p* > 0.05). Single‐task TUG, DT_motor_, DT Cognitive_forward_, and DT Cognitive_backward_ performances by groups are presented in Table 2.

**TABLE 2:** Single task TUG, DT_motor_, DT Cognitive_forward_, and DT Cognitive_backward_ performances by groups.

	P&HA group *n* = 30 median (min–max)	PoHA group *n* = 30 median (min–max) or mean ± SD	Control group *n* = 30 median (min–max) or mean ± SD	*p* ^a^	Pairwise comparison (*p*)
Single‐task TUG (s)	8.74 (5.36–16.07)	10.34 ± 2.95	6.41 (5.34–9.57)	< 0.001	< 0.001^b^ < 0.001^c^ 0.244^d^
DT_motor_ (s)	8.96 (6.19–16.81)	10.67 ± 3.08	6.79 ± 1.03	< 0.001	< 0.001^b^ < 0.001^c^ 0.270^d^
DTC (%)	4.60%	3.37%	3.68%		
DT Cognitive_forward_ (s)	9.03 (6.01–18.00)	9.20 (5.96–16.56)	6.47 ± 1.08	< 0.001	< 0.001^b^ < 0.001^c^ 0.710^d^
DTC (%)	3.80%	2.48%	1.08%		
DT Cognitive_backward_ (s)	8.76 (5.46–16.20)	11.18 ± 3.02	6.02 (4.99–9.80)	< 0.001	< 0.001^b^ < 0.001^c^ 0.031^d^
DTC (%)	0.10%	7.78%	3.80%		

Abbreviations: DT, dual task; DTC, dual‐task cost; P&HA, individuals with presbycusis using hearing aids; PoHA, individuals with presbycusis who do not use hearing aids.

^a^ Kruskal–Wallis test.

^b^ P&HA group—control group.

^c^ PoHA group—control group.

^d^ P&HA group—PoHA group.

## 4 Discussion

With aging, cognitive, physical, and sensory impairments occur in individuals. These impairments slow individuals' reflexes and movements, affecting their social functions and independent lives. In our study, we investigated the effect of hearing aids on nonauditory DT performance in elderly individuals with presbycusis. We included healthy adults and elderly individuals with presbycusis in our study. Changes in gait parameters (such as speed and stride length) are already expected in older adults due to aging. The main reason for including healthy adults in our study was to determine reference data in healthy adults when comparing the DT performance of older individuals with presbycusis who use and do not use hearing aids. In our study, the backward digit span and DT Cognitive_backward_ results of elderly individuals with presbycusis who used hearing aids were better than those of elderly individuals with presbycusis who did not use hearing aids but were still worse than healthy adults. In addition, there was a negative relationship between the severity of hearing loss and forward and backward digit span.

Elderly individuals with presbycusis have difficulty understanding speech in noisy environments. Therefore, more cognitive resources are allocated to the auditory task. In other words, individuals with hearing loss make extra effort in their auditory tasks and become tired (Dwarakanath and Manjula 2020). In addition, a cohort study (Lin et al. 2013) found that hearing loss independently caused cognitive decline. In the study, Modified Mini‐Mental State Examination and Digit Symbol Substitution were applied to elderly individuals with hearing loss, and the participants were followed for 6 years. The authors reported that individuals with hearing loss at baseline had a 24% increased risk of developing cognitive decline compared to those without. They also recommended that future studies investigate whether hearing‐correcting applications such as hearing aids prevent cognitive decline. Hearing aids improve individuals' communication skills by suppressing ambient sounds and amplifying speech sounds. Therefore, hearing aids are important in reducing listening efforts. However, the effect of hearing aids on nonauditory (e.g., visual) cognitive performance is unclear (Dwarakanath and Manjula 2020; van Hooren et al. 2005). Dwarakanath and Manjula (2020) investigated the relationship between hearing aid benefits, speech understanding ability in noise, and visual working memory (visual backward digit span) in elderly individuals with mild to moderate‐severe sensorineural hearing loss. The authors reported that older individuals who benefited better from hearing aids had better visual working memory. van Hooren et al. (2005) evaluated the memory, attention, executive functions and processing speed of 56 elderly individuals with hearing loss before and 12 months after applying a hearing aid fitting. Unlike the findings of Dwarakanath and Manjula, the authors reported no change in the individuals' cognitive functions. In our study, there was no difference in the educational status of elderly individuals in the P&HA and PoHA groups, and the SMME of all individuals was normal. We evaluated the participants' attention and short‐term memory with computerized forward and backward digit span tests. The audiovisual digit span test, the Wechsler Memory Scale‐Revised subtest, is part of a sizeable neuropsychological test battery (Karakaş et al. 2002). The audiovisual digit span test consists of four subtests that evaluate verbal and written responses to auditory or visually presented digit spans. In the original test, there is only forward recall of number sequences and no backward recall. However, the backward recall digit span test is also widely used in studies in the literature (Dwarakanath and Manjula 2020; Powell and Hiatt 1996). In addition, Powell and Hiatt (1996) reported that the backward visual digit span test is also applicable and better than the auditory application. For this reason, we applied the forward and backward visual digit span tests in our study. Since we wanted to evaluate visual cognitive skills rather than auditory cognitive skills, we did not apply auditory digit span in the study. Working memory is a system used for temporarily storing, managing, and manipulating information necessary to perform complex cognitive tasks. In the backward digit span test, participants are asked to remember the numbers and repeat them in reverse order. In this respect, the backward digit span test is more complicated and complex than the forward. In other words, while backward application mainly evaluates working memory, forward application evaluates short‐time memory. In our study, the better backward digit span results of individuals using hearing aids compared to those not using them demonstrate the positive effects of hearing aids on nonauditory cognitive functions and their role in reducing cognitive load. Our study also suggests the reasons for the contradictory findings in the literature (Dwarakanath and Manjula 2020; van Hooren et al. 2005). Accordingly, while there are no differences in simple cognitive tasks between elderly individuals who use and do not use hearing aids, there may be performance differences in more complex cognitive tasks. Storing, analyzing, and comprehending auditory information requires high levels of cognitive capacity. Unrehabilitated older individuals with presbycusis use their cognitive skills to discriminate and decode speech, not to process and store auditory information. This instantaneous perceptual‐cognitive effort may have led to changes in other nonauditory central structures and a permanent cognitive decline in the long term in elderly individuals with presbycusis who do not use hearing aids (Wong et al. 2010). In other words, our study shows that cognitive skills decrease as the severity of hearing loss increases and that unrehabilitated hearing loss in elderly individuals with presbycusis also affects nonauditory complex cognitive abilities.

Epidemiological studies have shown a strong association between presbycusis and the risk of cognitive impairment and dementia (Livingston et al. 2020). Various hypotheses have explained this association (Powell et al. 2022). The impaired auditory speech perception due to hearing loss leads to cortical cross‐modal reorganization in patients. In this process, the auditory cortex is functionally taken over by visual, somatosensory, or vibrotactile senses. This reorganization allows the auditory cortex to compensate for other sensory modalities in individuals with hearing loss (Glick and Sharma 2020). However, according to the sensory deprivation hypothesis, auditory neural deafferentation and cortical reorganization that develops to support other processes may cause atrophy in brain regions (Powell et al. 2022). Decreased grey matter density and temporal lobe volume have been observed in patients with hearing loss (Lin et al. 2014; Powell et al. 2022). In addition, a study reported that following hearing loss, there was increased activation of auditory (particularly in the right hemisphere), frontal, and pre‐frontal cortices during visual motion processing tasks. However, this reorganization was reversed within 6 months of hearing aid use (Glick and Sharma 2020). In this respect, our findings showing that individuals in the PoHA group have worse backward digit span results support the hearing loss‐dementia hypothesis from a clinical perspective. On the other hand, elderly individuals with hearing loss are particularly exposed to loneliness and social isolation. Loneliness and social isolation may cause poor cognitive functions through other psychological distress (Cardona and Andrés 2023). However, to better understand the relationship between hearing loss and cognitive decline, more longitudinal studies that also consider psychological effects are needed.

Although presbycusis negatively affects four main areas of quality of life (physical health, social relationships, psychological health, and environmental factors), it is often overlooked in routine health assessments (Eman 2017). This situation leads to insufficient awareness and insufficient evaluation of the effects of hearing loss on psychosocial and physical health problems (Eman 2017). Studies show that hearing aids contribute to quality of life by increasing hearing skills (Eman 2017). In addition, the current study observed that using hearing aids positively affects nonauditory cognitive skills in elderly individuals. Therefore, in the auditory rehabilitation process for elderly individuals, the cognitive benefits provided by hearing aids should also be considered, in addition to improving quality of life by increasing communication skills and social interaction. In other words, hearing aids support individuals' nonauditory cognitive skills beyond auditory perception and contribute to their independence in daily life activities.

Walking occurs due to the integrated work of sensory balance systems and the musculoskeletal system. In addition, increasing evidence shows that hearing significantly impacts walking performance (Gopinath et al. 2016; Wollesen et al. 2018). Hearing loss can affect individuals' ability to walk in two ways. The first is that the auditory and vestibular end organs are located in the same bony labyrinth. Both organs show phylogenetic and anatomical similarities. For this reason, some hearing losses may affect the vestibular organ (Melo et al. 2018). According to this hypothesis, balance and gait disorders can be detected by simple single‐task balance tests such as standing on one leg or walking (Melo et al. 2018). The other hypothesis is that individuals create three‐dimensional spatial hearing maps of the environment with monoaural and binaural cues and use them to determine their location according to statically and dynamically changing sound sources (Campos, Ramkhalawansingh, and Pichora‐Fuller 2018; Eddins and Hall 2010). The improvement in single‐task balance skills of individuals when auditory cues are presented confirms the spatial auditory map hypothesis (Gandemer et al. 2017). However, in daily life, balance and walking functions mostly occur in the form of DT, and single‐task balance assessments can be misleading in assessing functionality. Attention is important to ensure coordination during DT walking, and the importance of attention processes such as alertness, selective attention, sustained attention, and divided attention increases (Hirano et al. 2020). Wollesen et al. (Wollesen et al. 2018) evaluated the DT walking performance of 73 elderly individuals with and without hearing loss. The authors reported that DT walking deteriorated as hearing loss increased. Bruce et al. (2019) applied cognitive and balance tasks simultaneously under DT to elderly individuals with normal hearing, with presbycusis, and healthy young individuals. The authors stated that young individuals can allocate their attention to easier tasks, but older individuals with presbycusis primarily use their attention to maintain their posture. We did not find any literature study investigating the performance of DT in elderly individuals using hearing aids. Our findings were similar to those of Bruce et al. In other words, the DT performance of healthy adults was better than that of older adults. In addition, the DT cognitive backward performance of elderly individuals using hearing aids was better than those who did not use hearing aids. During DT, attention is divided (Statistics Canada 2006). This situation can be explained by the fact that the PoHA group has worse working memory. In other words, individuals with presbycusis who do not use hearing aids may have difficulty allocating their limited cognitive capacity to tasks. Therefore, elderly individuals who do not use hearing aids are more vulnerable to falls due to insufficient cognitive capacity, especially in challenging cognitive DT situations. An epidemiological study conducted in Canada supports this finding (Chien and Lin 2012). Individuals with hearing loss experience mobility and agility problems (65%) rather than communication difficulties (12%) (Statistics Canada 2006). Falls, which are especially common in elderly individuals, create an additional burden on public health systems and cause permanent disabilities and fatal injuries in elderly individuals.

In conclusion, our study indicates that the use of hearing aids is important not only for enhancing communication skills and reducing listening effort but also for supporting nonauditory cognitive functions, minimizing the risk of falls during DT, and improving overall quality of life. However, despite all these obligations, the prevalence of regular hearing aid use (14.2% of individuals aged 50 and over) is very low (Chien and Lin 2012). This may be due to hearing aids not being ergonomic or the harms of hearing loss being unknown. Therefore, more R&D investments are needed to produce ergonomic and affordable hearing aids, and more awareness campaigns are required to popularize hearing aids, especially in elderly individuals.

This study has some limitations. The sample size in the present study is relatively small. In the future, the effect of hearing aids on nonauditory cognitive functions could be investigated with a larger sample group. In addition, although the P&HA group demonstrated better performance on the backward digit span test and DT cognitive backward performance compared to the PoHA group, the reasons for these differences have not been fully explained. Future research should include longitudinal studies to gain a better understanding of the underlying causes of these differences.

## 5 Conclusion

Healthy adults have better single‐task and DT performance than older individuals. Also, elderly individuals with presbycusis who use hearing aids have better visual working memory and better DT performance in challenging cognitive tasks than elderly individuals who do not use hearing aids. These findings indicate that the use of hearing aids and their proper fitting are important not only for improving communication skills and reducing listening effort, but also for supporting nonauditory cognitive functions, minimizing the risk of falls during dual‐tasking, and enhancing overall quality of life.

## Author Contributions

**Emre Soylemez**: writing–review and editing, visualization, writing–original draft, investigation, conceptualization, methodology, validation, funding acquisition. **Tugce Gurel Soylemez**: formal analysis, project administration, data curation, resources, writing–original draft, investigation, conceptualization, writing–review and editing. **Aydin Sinan Apaydin**: supervision, conceptualization, investigation, validation, methodology. **Zuhal Koc Apaydin**: supervision, resources, writing–review and editing, methodology. **Murat Yasar**: methodology, conceptualization, visualization, writing–review and editing, project administration, formal analysis, data curation.

## Ethics Statement

We obtained both verbal and written consent from all participants in accordance with the Declaration of Helsinki. Ethical approval was obtained for the study from the university's clinical research commission (2024‐KAEK‐28).

## Consent

Written and verbal consent was obtained from the participants.

## Conflicts of Interest

The authors declare no conflicts of interest.

### Peer Review

The peer review history for this article is available at https://publons.com/publon/10.1002/brb3.70114.

## Data Availability

The data that support the findings of this study are available on request from the corresponding author. The data are not publicly available due to privacy or ethical restrictions.
